# Editorial: Gravitational influence on human cognition and behavior: emerging research

**DOI:** 10.3389/fpsyg.2026.2008421

**Published:** 2026-10-23

**Authors:** Jeremie Gaveau, Shinji Yamamoto, Keisuke Tani, Keisuke Kushiro, Elisa Raffaella Ferre

**Affiliations:** 1 INSERM U1093-CAPS, Université Bourgogne Europe, UFR des Sciences du Sport, Dijon, France; 2 Graduate School of Sport Sciences, Nihon Fukushi University, Mihama-cho, Japan; 3 Faculty of Psychology, Otemon Gakuin University, Ibaraki, Japan; 4 Graduate School of Human and Environmental Studies, Kyoto University, Kyoto, Japan; 5 School of Psychological Sciences, Birkbeck, University of London, London, United Kingdom

**Keywords:** adaptation, behavior, cognition, countermeasure, gravity, microgravity, sensorimotor

Human perception and action evolved under an environmental regularity so constant that it is easily overlooked: Earth's gravity. Gravity loads the body, but it also provides a reference for spatial orientation and a prior for predicting how bodies and objects move (Tagliabue and McIntyre, 2011; Lacquaniti et al., 2013; Gaveau et al., 2016; Tani et al., 2020; Gallagher et al., 2021; Yamamoto et al., 2026). Vestibular signals become multisensory at early processing stages and are integrated with visual, proprioceptive, somatosensory, and visceral information (Lackner and DiZio, 2005; Angelaki and Cullen, 2008; Lacquaniti et al., 2015). The nine contributions examine altered, absent, or simulated gravity through complementary experimental, computational, and theoretical approaches. Across them, a shared view emerges: altered gravity challenges predictions calibrated through lifelong experience at 1G. Topic contributions are linked by author name; broader literature is cited conventionally.

## From sensory mismatch to adaptation

Maillard and Bresciani synthesize how hypogravity reduces the reliability of otolithic, proprioceptive, and load-related somatosensory cues, increases reliance on vision, and perturbs verticality perception, balance, and manual control. Jahanian Najafabadi and Kroger extend this multisensory account to time perception: gravity-congruent motion supports temporal prediction, whereas real or simulated alterations of gravitational information can distort duration judgments. These findings accord with evidence that gravity is incorporated into internal representations used for perception and movement planning (Indovina et al., 2005; Gaveau and Papaxanthis, 2011; Laurens et al., 2013; Lacquaniti et al., 2015; Gaveau et al., 2016, 2021; Cullen, 2019; White et al., 2020). When current sensory evidence no longer matches predictions shaped at 1G, sensory conflict and prediction error arise, engaging multisensory reweighting and recalibration (Gaveau et al., 2011, 2016; Senot et al., 2012; Carriot et al., 2015, 2021; Laurens and Angelaki, 2017; Tani et al., 2025; Bonnard et al., 2026). This sequence is summarized in Figure 1.

**Figure 1:**
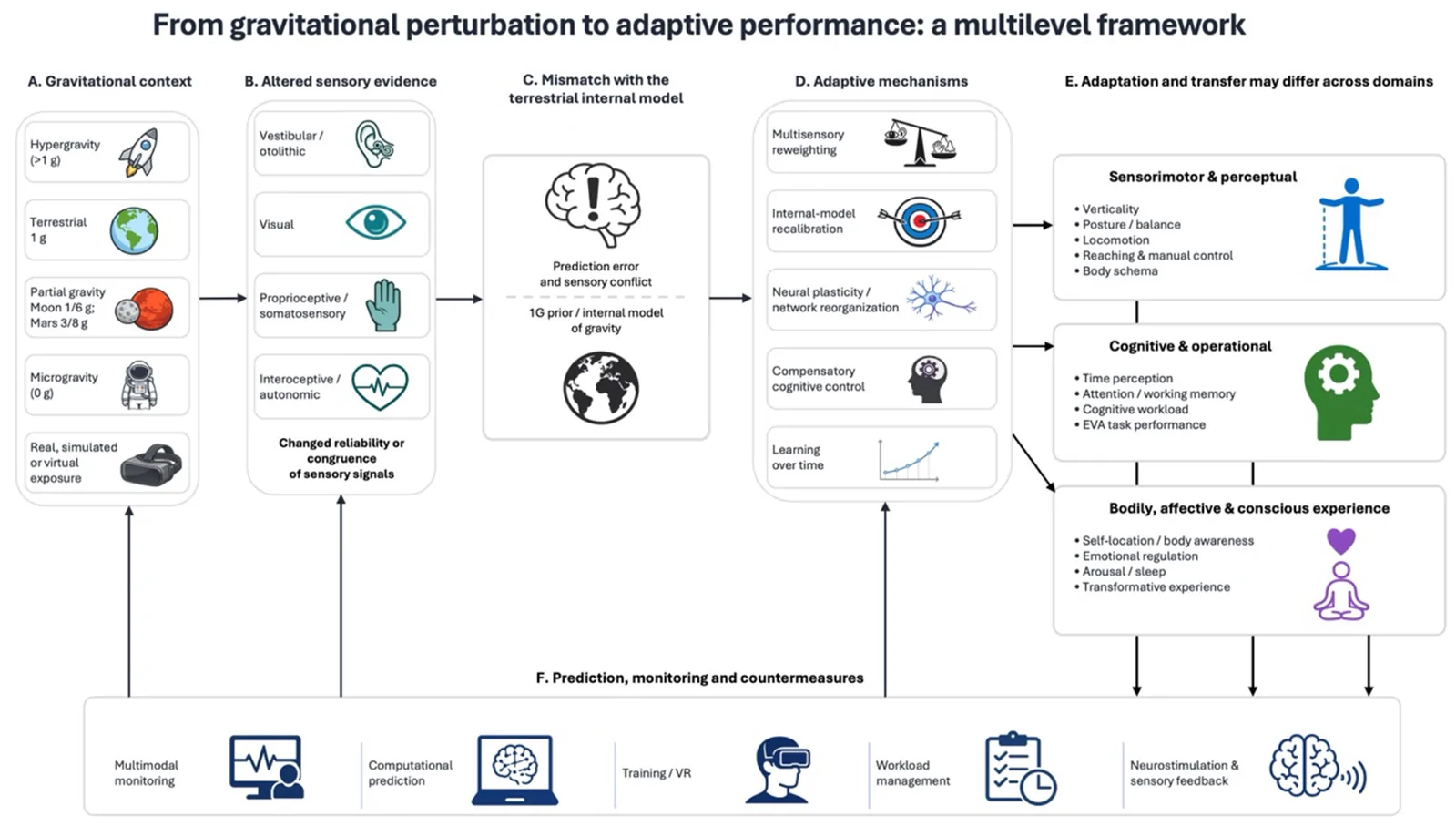
From gravitational perturbation to adaptive performance: a multilevel framework. (**A)** Changes in gravitational context, including hypergravity, terrestrial gravity, partial gravity, microgravity, and simulated or virtual exposure, **(B)** alter the reliability and congruence of vestibular, visual, proprioceptive, somatosensory, interoceptive, and autonomic signals. **(C)** When these signals diverge from predictions based on the terrestrial 1G prior, prediction errors and sensory conflict emerge. **(D)** These mismatches may engage complementary adaptive mechanisms, including multisensory reweighting, recalibration of internal models, neural plasticity and network reorganization, compensatory cognitive control, and learning over time. **(E)** Their consequences unfold across partly dissociable domains: sensorimotor and perceptual function, cognitive and operational performance, and bodily, affective, and conscious experience. Adaptation and transfer may therefore differ across functional systems. **(F)** Multimodal monitoring, computational prediction, virtual-reality training, workload management, neurostimulation, and sensory feedback can be used to assess, facilitate, and individualize adaptation. Information obtained from behavioral, cognitive, physiological, and experiential outcomes can guide the design of training, exposure protocols, and countermeasures.

Scaramelli et al. provide a neural counterpart to this account. Their coordinate-based meta-analysis of 15 neuroimaging studies identified convergent changes in a right-lateralized network spanning the precentral and postcentral cortices, insula, and operculum—regions central to vestibular-somatosensory integration, spatial orientation, and motor control. Rare complementary ISS recordings by Cebolla et al. (2016) showed enhanced alpha–mu desynchronization during weightlessness and source-level involvement of motor, vestibular, and cerebellar regions, consistent with increased postural and multisensory demands. Together, these findings support network reorganization during adaptation without implying uniformly beneficial plasticity. Rock et al. add a key qualification. After blindfolded jumping in simulated hypogravity, participants showed clear motor aftereffects but no transfer to a visual gravity-judgment task. Vision was therefore unnecessary for motor adaptation yet may have been necessary for cross-domain transfer. Adaptation is thus distributed, sensory-dependent, and only partly generalizable.

## Consequences across partly dissociable domains

As summarized in Figure 1, adaptation may affect sensorimotor and perceptual, cognitive and operational, and bodily, affective, and conscious domains. Evidence is strongest for sensorimotor and perceptual functions, including verticality, posture, locomotion, reaching, manual control, and body representation, but may extend to cognitive and socio-affective functioning (Arshad and Ferrè, 2023). The review by Jahanian Najafabadi and Kroger connects gravity to temporal cognition through visual motion, vestibular input, autonomic state, and sensorimotor prediction. Outside this Research Topic, Salatino et al. (2021) similarly showed that otolith unloading during parabolic flight enhanced stimulus-driven attentional capture while weakening voluntary maintenance of attention.

Anderson et al. address the operational expression of these cognitive demands. In an integrated virtual-reality (VR) lunar extravehicular-activity simulation, increasing geological-task complexity raised subjective workload, impaired working-memory performance and the quality and efficiency of sample selection, and altered cardiac responses. Because physical gravity remained at 1G, these results do not demonstrate a gravity-induced decrement. Rather, they show why cognitive workload must be incorporated into models of performance during future lunar and Martian operations. Bonarrigo et al. show that operationally relevant VR tasks elicit greater neural activation than matched microcognitive corollary tasks, supporting VR as an ecologically valid platform for probing spaceflight-relevant cognitive engagement. Whether such increased activation translates into beneficial training or an effective countermeasure remains to be established. At the same time, VR-based approaches should consider whether repeated virtual exposure may alter the processes being assessed or trained. Yamamoto et al. (2026), for example, revealed that although gravity prior remained stable in VR, its precision gradually declined across trials.

Two contributions open a more exploratory dimension. Nezami and Ferre propose that terrestrial gravity functions as a deeply established “1G super-prior,” whose disruption may alter bodily coherence, self-location, and conscious experience. Kent's “mental gravity” framework considers how verticality, balance, weight, and spatial direction may scaffold emotional representation and non-clinical mental-health promotion. These perspectives extend gravitational neuroscience toward affect and consciousness, but their proposed mechanisms remain less directly established than the sensorimotor effects summarized above. Collectively, the papers therefore support a multilevel account while emphasizing that adaptation and transfer may differ across sensory modalities, tasks, timescales, and functional domains.

## From characterization to prediction and countermeasures

These contributions also point toward prediction and intervention. Fricke et al. propose transcutaneous auricular vagus nerve stimulation as a portable countermeasure targeting cognition, autonomic regulation, and sensorimotor integration; its efficacy in altered gravity remains to be tested. Progress will require standardized and longitudinal protocols, explicit distinction between real, mechanically simulated, and visually simulated gravity, and multimodal monitoring capable of capturing individual trajectories of disruption, adaptation, stabilization, and re-adaptation. Figure 1 therefore frames prediction, monitoring, and countermeasures as a feedback layer that can inform and potentially shape adaptation across these domains.

## Conclusion

Taken together, the contributions to this Research Topic show that gravity is not merely a mechanical force acting on the body, but a pervasive reference signal incorporated into the brain's representations of the body and environment. When gravitational information changes or becomes unreliable, mismatches arise between current sensory evidence and predictions shaped by terrestrial gravity, engaging multisensory reweighting, internal-model recalibration, and neural reorganization. These adaptive processes unfold across sensorimotor, perceptual, and cognitive systems, and may also extend to bodily, affective, and conscious experience. Crucially, adaptation is neither instantaneous nor uniform across domains. Understanding this variability may be essential for predicting individual responses, preserving operational performance, and developing effective monitoring, training, and countermeasures for future human exploration beyond Earth.
